# Iron Mediates Radiation-Induced Glioblastoma Cell Diffusion

**DOI:** 10.3390/ijms26104755

**Published:** 2025-05-16

**Authors:** Stephenson Boakye Owusu, Akalanka B. Ekanayake, Alexei V. Tivanski, Michael S. Petronek

**Affiliations:** 1 Department of Radiation Oncology, Division of Free Radical and Radiation Biology, University of Iowa, Iowa City, IA 52242, USA; 2 Department of Chemistry, University of Iowa, Iowa City, IA 52242, USA

**Keywords:** glioblastoma, ionizing radiation, iron, ferritin overexpression, tumor migration

## Abstract

Radiation therapy is a standard of care treatment for patients with glioblastoma. However, patients’ survival rate is dismal, with nearly all patients experiencing disease progression after treatment. Enriched iron content associated with increased transferrin receptor (TfR) expression is an indicator of poor glioblastoma patient outcomes; however, the underlying contributions to tumor progression remain elusive. The goal of this present study is to understand how iron metabolism in glioma contributes to radiation-induced glioblastoma cell motility. U251 and a doxycycline-inducible ferritin heavy chain overexpressing U251 (U251 FtH^+^) cell line were used. For in vitro studies, cells were irradiated with 2 Gy using a ^37^Cs source, and after 72 h, atomic force microscopy (AFM) nanoindentation was employed to assess changes in cell stiffness following irradiation. Cell motility was studied using temporal confocal microscopy. For in vivo studies, U251 cells were grown in the rear flanks of female nude athymic mice, and the tumor was irradiated with five fractions of 2 Gy (10 Gy). The tumors were then imaged using a GE 7T small animal MRI to assess changes in T2* MRI, and colorimetric analysis of labile iron was performed using ferrozine. Following irradiation, a biomechanical shift characterized by decreased cell stiffness along with increased cell motility occurred in U251 cells, which corresponded to increased TfR expression. FtH overexpression completely reversed the enhanced cell motility following irradiation. Irradiation of U251 tumors induced the same iron metabolic shift. Interestingly, the change in labile iron in U251 tumors corresponded with an increase in T2* relaxation times, suggesting that T2* mapping may serve as a surrogate marker for assessing radiation-induced changes in iron metabolism.

## 1. Introduction

Glioblastoma (GBM) is the most common and aggressive primary brain malignancy in adults [1]. Maximal safe surgical resection and radiation therapy in combination with temozolomide is the standard of care treatment regimen for GBM tumors [2,3,4]. Despite this rigorous treatment approach, patients’ survival rate is poor, with a median overall survival of 14.6 months and progression-free survival of 6–7 months [5]. Nearly all GBM patents (≥95%) will experience disease progression. However, the disease progression process is still poorly understood.

Iron accumulation is a common feature of aggressive tumors [6] and consistent with this posit, it has been previously established that glioblastoma tumors have increased iron content along with increased expression of transferrin receptor (TfR) and ferritin heavy chain (FtH) relative to normal brain tissue, which is associated with worse clinical outcomes [7,8,9]. However, the mechanism(s) by which iron can facilitate tumor growth are still elusive. Recently, it has been discovered that isocitrate dehydrogenase mutant glioma cells that accumulate excess iron are significantly less stiff, and ferrous iron is able to exacerbate increased membrane fluidity and cell motility associated with this phenotype [10]. Thus, it can be hypothesized that at least one role for iron in promoting glioma cell aggression is through biomechanical alterations that facilitate cell motility.

While radiation therapy is the current standard of care for GBM, paradoxically, radiation alone can contribute to glioblastoma tumor progression by inducing a tumor evolution following initial treatment [11]. These oxidative stress-mediated adaptive responses include innate immune activation, hypoxia-associated metabolic shifts, and restructuring of the extracellular matrix (ECM) to facilitate motility [12,13,14,15]. In cells, redox-active (i.e., labile) iron is considered a major determinant of cellular responses to oxidative stress. Labile iron catalyzes oxidative damage through reactions with H_2_O_2_ and molecular oxygen to generate HO^•^ or O_2_^•−^, respectively [16]. Studies have shown that iron accumulation and iron regulatory proteins, including transferrin (Tf), transferrin receptor (TfR), and ferritin heavy chain (FtH), increased after radiation treatment in breast cancer [17]. However, the influence of iron metabolic adaptations following radiotherapy and the role of iron as a contributor to tumor progression have yet to be thoroughly explored. Therefore, the overarching goal of this study was to interrogate the role of iron as an underlying feature of the biomechanical adaptation of GBM cells caused by ionizing radiation.

## 2. Results

### 2.1. Radiation-Induced Iron Accumulation Enhances Glioma Cell Migration

**Figure 1:**
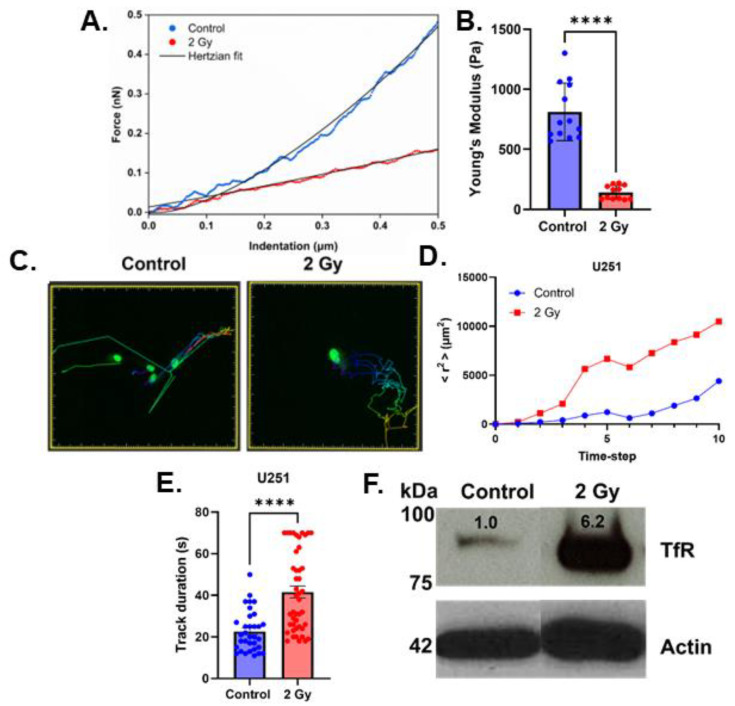
Radiation-induced iron accumulation enhances cell motility. (**A**,**B**) Atomic force microscopy analysis of U251 cells 72 h after receiving 2 Gy radiation. Representative force–indentation distance plots (approach to the cell surface data only) for control (blue) and 2 Gy (red); black solid lines are corresponding fits using the Hertzian elastic contact model (**A**). Young’s modulus values for individual cells (**B**). Error bars represent mean ± SEM with **** *p* < 0.00001 using Welch’s *t*-test. (**C**) Representative displacement maps of U251 cells treated with 2 Gy of irradiation and incubated for 72 h. (**D**) Mean squared displacement (<r^2^>) over time (right). Each time step represents a 15 min interval. (**E**) Average cell motility duration in U251 cells (treated with 2 Gy of irradiation and incubated for 72 h) measured for 18 h. Error bars represent mean ± SEM with **** *p* < 0.00001 using Welch’s *t*-test. (**F**) Western blot analysis of transferrin receptor (TfR) expression. β-actin was used as a loading control.

Recently, it has been shown that the accumulation of iron in isocitrate dehydrogenase mutant glioma lowers cell stiffness and enhances membrane fluidity and cell migration [10]. Thus, iron accumulation may play an important role in the biomechanical properties associated with tumor migration. Also, it has previously been reported that radiation can enhance cell motility [18,19] and that oxidant production can continue to increase up to 72 h following irradiation [20]. Thus, for this current study, cells were irradiated with 2 Gy and left to adapt for 72 h. Consistent with the hypothesis that there are biophysical changes following irradiation, a significant decrease in Young’s modulus was observed (Figure 1A,B). Following this observation, confocal microscopy was used to perform live cell tracking on cells 72 h post-irradiation, and the calculation of mean square displacement over time showed that irradiated cells have an accentuated mean squared displacement over time, an indication of increased cell motility (Figure 1C,D). Moreover, the irradiated cells showed a significant increase in track duration over the course of 18 h (Figure 1E), meaning that irradiated cells have a greater propensity to stay in motion. Consistent with a previous report that TfR expression inversely correlates with Young’s modulus [10], irradiated cells have significantly higher levels of TfR expression (Figure 1F). Taken together, these results suggest that the radiation-enhanced labile iron of iron metabolism may underlie the biomechanical changes associated with increased cell motility.

### 2.2. Radiation Increases MRI Detectable Labile Iron In Vivo

**Figure 2:**
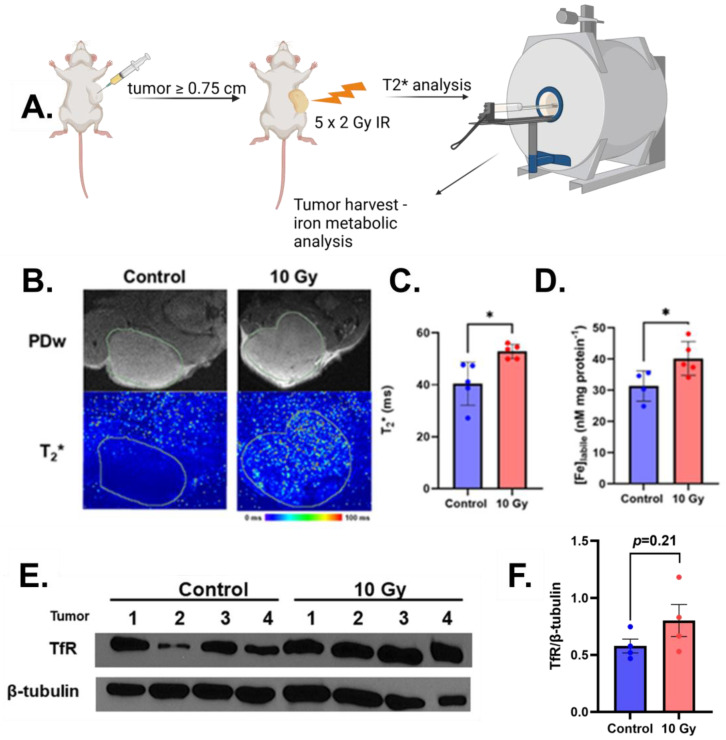
Radiation increases MRI-detectable labile iron in vivo. (**A**) General mouse treatment study scheme. U251 tumor-bearing mice were irradiated with 2 Gy for 5 consecutive days (10 Gy total). (**B**,**C**) Immediately following the final fraction of radiation, prior to euthanasia and tissue harvest, mice were imaged on a 7T GE small-animal MRI. Representative proton-density-weighted MRI for tumor delineation (top panel, green contour indicates tumor region of interest) and T2* maps. (**B**) Mean T2* relaxation times in the tumor (**C**). Mean T2* relaxation times were evaluated using the Label Statistics package within the Slicer3D software package. Error bars represent mean ± SEM with * *p* < 0.05 using Welch’s *t*-test. (**D**) Relative labile iron concentrations in tumor tissues were evaluated using the ferrozine colorimetric assay. Error bars represent mean ± SEM with * *p* < 0.05 using Welch’s *t*-test. (**E**) Western blot analysis of transferrin receptor (TfR) expression with β-tubulin was used as a loading control. Each lane represents a tumor of an individual animal (i.e., *n* = 4 individual tumors). (**F**) Band quantification of Western blot. Error bars represent mean ± SEM (*n* = 4) using Welch’s *t*-test.

Following the intriguing initial in vitro studies, we aimed to evaluate the biological relevance by studying the effects of ionizing radiation on intratumoral iron content in a subcutaneous tumor xenograft model. U251 cells were subcutaneously inoculated and allowed to grow to ≥0.75 cm in any direction to have a large enough mass for tissue analysis. The tumors were then irradiated with 10 Gy (fractionated 2 Gy successively for 5 days) to mimic a conventional radiotherapy treatment (Figure 2A). Previously, it has been reported that T2* MRI can detect iron metabolic changes in GBM subjects [21,22]; thus, we interrogated T2* changes immediately following the completion of the final fraction of radiation. Irradiated tumors showed a significantly higher T2* relaxation time (Figure 2B,C), suggesting increased relative iron content [21,23]. Consistent with this purported change, there was a significant increase in redox-active iron in the irradiated tumors compared to the non-irradiated tumors (Figure 2D). Furthermore, immunoblotting analysis of lysed tumor tissue revealed an enhanced expression of TfR that was previously observed in vitro (Figure 2E). Together, these results demonstrate that radiation enhances relative iron content in GBM tumors and can be detected using MRI.

### 2.3. Ferritin Overexpression Reverses Radiation-Induced Cell Motility

**Figure 3:**
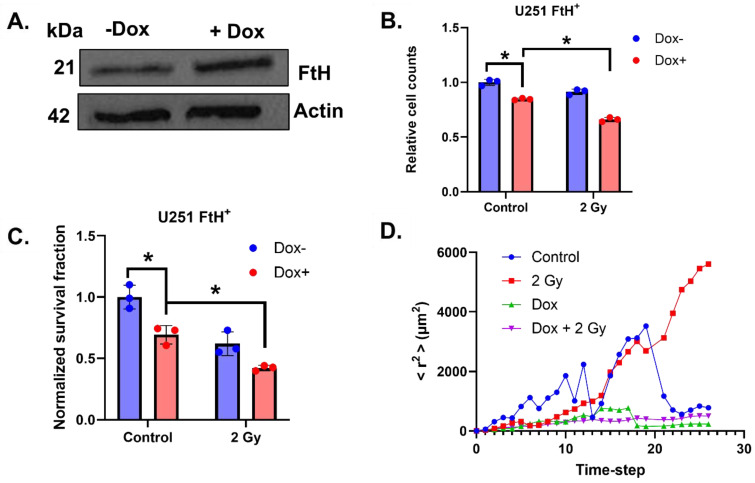
Ferritin overexpression reverses radiation-induced cell motility. (**A**) Western blot validation of FtH overexpression following 72 h of 1 µg mL^−1^ doxycycline treatment given daily (total load = 3 µg mL^−1^). (**B**,**C**) Relative cell counts (**B**) and colony formation (**C**) in U251 FtH+ cells 72 h following 2 Gy irradiation ± 1 µg mL^−1^ doxycycline treatment given daily (total load = 3 µg mL^−1^). For colony formation, cells were re-plated as single cells without the addition of doxycycline and allowed to expand freely for 7 days. Error bars represent mean ± SEM (*n* = 3) with * *p* < 0.05 using a two-way ANOVA test. (**D**) Mean squared displacement (<r^2^>) over time for the total cell populations (nmin = 100 cells). Cells were counted and re-plated as single cells on glass-bottom 6-well plates for confocal analysis.

To verify that the observed phenotypes are due to iron metabolic adaptations, a previously established doxycycline-inducible FtH overexpression model (U251 FtH^+^), which is capable of absorbing excess intracellular redox-active iron, was utilized [21]. Overexpression was confirmed by treating cells with 1 µg mL^−1^ doxycycline daily for 72 h (total drug load = 3 µg mL^−1^) and evaluating FtH overexpression via Western blotting (Figure 3A). The effects of FtH overexpression on standard cell growth metrics (growth rate and colony formation) were evaluated, which revealed that 72 h of FtH overexpression significantly impaired cell growth and colony formation (Figure 3B,C), which is consistent with a previous report in non-small cell lung cancer [24]. Consistent with the overarching hypothesis that radiation can stimulate iron-dependent growth, the effects of FtH overexpression were exacerbated in cells treated with 2 Gy irradiation 72 h prior to experimentation. These results are an indication that iron accumulation can play an underlying role in radiation adaptations that modulate cell growth. To further substantiate our in vitro observations that biomechanical remodeling by radiation is associated with the observed iron metabolic shift, we evaluated cell motility using this genetically engineered system 72 h following irradiation. Interestingly, in U251 FtH^+^ cells, there is no observable difference in mean squared displacement over time, but the irradiated cells appear to continue in motion longer than the control cells (Figure 3D). There were no observable differences in mean squared displacement in cells following the induction of FtH overexpression with or without irradiation. This is owed largely to the fact that FtH overexpression by itself appears to impair cell motility, as evidenced by the minimal increase in mean squared displacement over time, which limits the ability to detect a difference between irradiated and unirradiated cells. Thus, it appears that in general, iron availability may be a critical feature of glioma cell motility.

## 3. Discussion

With the poor clinical responses associated with GBM tumor progression, there is a dire need for a more robust understanding of the fundamental mechanisms that drive this process to generate better patient outcomes. While radiotherapy is a standard therapy for glioma tumors, many reports have shown that irradiation induces a pro-migratory effect on glioma cells; however, there is no clear report on what underlies such an effect [25,26,27]. Ionizing radiation is known to generate excess reactive oxygen species, which can accumulate in cell culture up to 72 h following radiation exposure [20]. A recent report has shown that enhanced oxidative stress can promote GBM cell motility [28]. Many studies have reported that pro-migratory effects of radiation on glioma tumors could be due to extracellular matrix (ECM) stiffness [29,30,31,32]. However, our findings also indicate that not only does radiation regulate ECM stiffness, but it can also cause biomechanical remodeling by decreasing stiffness at the cellular level. Moreover, radiation also stimulates GBM cell motility 72 h after radiation exposure, which corresponds with a robust increase in TfR expression. Free iron is capable of catalyzing oxidations; thus, there is no doubt that iron could modulate such properties [33]. This posit is consistent with the recent report that iron accumulation can promote the migration of isocitrate dehydrogenase (IDH) mutant glioma cells, congruent with increased TfR and decreased cell stiffness [10]. The correlation between the pro-migratory biomechanical properties of GBM cells (e.g., decreased cell stiffness) and enriched TfR expression may have real-world, clinical implications, as it has been shown that enhanced TfR expression is associated with poor clinical outcomes [7,34]. Thus, the radio-modulation of TfR expression may serve as a biomechanical regulatory feature to promote GBM progression.

Moreover, the proposed iron-dependent tumor growth and motility induced by radiation was causally validated using FtH overexpressing U251 cells. FtH overexpression alone was able to impair GBM cell growth and colony formation, an effect that has been previously observed in non-small cell lung cancer [24,35]. A similar inhibition of growth by FtH overexpression was observed in U251 cells; however, this effect was exacerbated following radiation, further supporting the hypothesis that radiation can promote iron-dependent growth. Furthering our hypothesis that this effect may be associated with iron-dependent cell motility, FtH overexpression was able to completely reverse radiation-enriched cell motility. Interestingly, this effect of FtH overexpression on cell motility was not able to be differentiated between irradiated and non-irradiated cells because FtH overexpression alone completely blunted cell motility. Changes in biomechanical properties of cells, such as membrane fluidity and cell stiffness, are often related to cell motility, leading to tumor growth and metastasis [36,37,38,39], and, hence, our findings indicate that redox-active iron could play a central role in this process. This would be congruent with a recent translational study, which showed that GBM progression can be driven by ferroptosis induction [40]. In addition, gallium-based therapy as a redox-inactive iron mimic has shown promise as a means to enhance GBM radiation responses [41,42]. Thus, gallium-based therapeutics may be more efficacious in the adjuvant setting to exploit the iron metabolic shift caused by radiation. Therefore, the identification of a unique iron-dependent phenotype associated with radiation opens the door for a myriad of novel exploratory avenues, including the role of iron as a key biomechanical regulatory feature, as well as further evaluation of iron-dependent therapeutic approaches to exploit radiation-induced tumor adaptations as a potential therapeutic vulnerability.

Importantly, the radio-modulation of iron metabolism was retained in vivo. Using a conventional radiation fractionation scheme of daily 2 Gy fractions up to a 10 Gy total dose, a notable increase in TfR expression was observed that was coincident with a significant increase in labile iron. These results are consistent with previous reports that isocitrate dehydrogenase mutant glioma cells grown in vivo have a significant growth rate with a significant increase in TfR expression as compared to the wild-type counterpart [10]. From a translational science perspective, the iron metabolic shift that occurred during radiotherapy was able to be detected using T2* mapping. Previously, it has been reported that T2*-based MRI exhibits iron oxidation state specificity, which allows it to detect increases in labile iron and can serve as a prognostic marker in GBM patients [21]. Therefore, monitoring iron metabolic changes with T2* mapping may serve as a useful tool to monitor and possibly predict iron-driven GBM tumor progression.

## 4. Materials and Methods

### 4.1. Cell Culture

U251 glioma cells (Millipore Sigma (Burlington, MA, USA), 09063001) and U251 FtH^+^ cells (lentiviral transduction protocol provided in [21]) were cultured in DMEM-F12 media (15% FBS, 1% penicillin-strep, 1% Na-pyruvate, 1.5% HEPES, 0.1% insulin, and 0.02% fibroblast growth factor) and grown to 70–80% confluence at 21% O_2_. Cell lines were authenticated prior to use. For all in vitro experiments, cells were irradiated at 2 Gy using a ^37^Cs source and incubated at 37 °C for up to 72 h prior to experimentation.

### 4.2. Colony Formation

Cells were harvested via trypsinization, resuspended, and plated as single cells (400 cells). Cells were allowed up to 10 days to form colonies (≥50 cells) before being stained. For analysis, cells were fixed with 70% ethanol, stained with Coomassie blue, and the number of colonies was counted manually under a microscope. The plating efficiency for each group was calculated using the following formula:

$$Plating\;efficiency\left(\%\right)=\left(\frac{\#\;colonies\;counted}{\#\;cells\;plated}\right)*100$$

The plating efficiency from each group was normalized to their respective control to generate a normalized survival fraction.

### 4.3. Cell Stiffness

Cells were irradiated at 2 Gy and incubated at 37 °C for 72 h, after which they were trypsinized and counted. Cells were then plated as single cells (≈10,000 cells) on a cover slip in a 60 mm^3^ dish and allowed to adhere overnight prior to analysis. All cell stiffness measurements were performed using a Molecular Force Probe 3D AFM (Asylum Research, Santa Barbara, CA, USA). Nanoindentation measurements used AFM tips (Nanotools, Munich, Germany, biosphere™ B2000-CONT) with a high-density, diamond-like carbon sphere with a radius of 2 µm attached at the end of a flexible cantilever with a spring constant of 0.2 N/m. Single cells were located using an AFM optical camera before the nanoindentation measurements. Nanoindentation measurements involved collecting force–vertical displacement curves in contact mode in a phosphate-buffered saline buffer at 22 ± 2 °C at an approximate center of each individual cell with a maximum applied loading force of 3 nN and a 500 nm/s AFM tip approach velocity. At least three repeated force–vertical displacement measurements were collected per cell, and at least 12 individual cells were measured per sample. The force–vertical displacement profiles were converted to force–indentation distance, and the approach to the surface data was fit using the Hertzian elastic contact model to calculate the corresponding cell stiffness (Young’s modulus) as described previously [10,43]. The AFM tip was modeled as a sphere with a radius of 2 µm, and Poisson ratios of the tip and cell were assumed to be 0.2 and 0.25, respectively. For each cell, the fitted Young’s modulus results based on at least three repeated measurements were averaged to yield an average cell stiffness value.

### 4.4. Live Cell Motility

Cells were irradiated at 2 Gy and incubated at 37 °C for 72 h, after which they were trypsinized and counted. Cells were then plated as single cells (≈8000 cells) in a glass-bottom 6-well plate 24 h prior to analysis. Prior to analysis, cells were stained for at least 3 h with NucSpot^®^ 488 Live Cell stain, as per the manufacturer’s instructions (Biotium #40081, Freemont, CA, USA). Live cells were imaged with a Zeiss LSM 980 AiryScan2 (Pleasanton, CA, USA) confocal microscope temporally in 15 min intervals to evaluate cell motility. Image analysis was performed using Oxford Imaris 10.2 to calculate mean squared displacement over time.

### 4.5. Western Blotting

Western blotting was performed and was slightly modified from [41]. Briefly, cells and tissues were lysed in a RIPA buffer (Sigma Aldrich, St Louis, MO, USA), and the total protein of the supernatant was quantified using a DC™ protein assay kit (Bio-Rad, Hercules, CA, USA). Proteins (20 μg each) were then separated by 4–15% SDS-PAGE electrophoresis (Bio-Rad) and transferred to 0.22 μm pore size PVDF membranes (Bio-Rad) at 25 V-2.5 A for 10 min using Trans Blot turbo (Bio-Rad). The membranes were then blocked with the EveryBlot Blocking buffer (Bio-Rad) for 5 min and incubated at 4 °C overnight with either anti-transferrin receptor (1:1000, Proteintech-17435-1-AP, Rosemont, IL, USA), anti-β-tubulin (Proteintech-66240-1-Ig)and anti-β-actin (Proteintech-66009-1-Ig). Membranes were washed 3× for 5 min each with 1× TBST (Bio-Rad) and were incubated with either goat anti-mouse (1:5000, Cell Signaling, Danvas, MA, USA) or anti-rabbit (1:5000, Cell Signaling, Danvas, MA, USA) and conjugated with HRP for 1 h at room temperature. After 10 min of washing the membranes with 1× TBS-T (3×), the signals were developed with a chemiluminescent kit (Super Signal West Pico & Super Signal West Femto, Thermo Scientific, Waltham, MA, USA) and exposed on an X-ray film (Research Products International, Cleveland, OH, USA). The same approach was used to analyze protein expression in harvested tumors from individual mice (*n* = 4).

### 4.6. In Vivo Studies

U251 cells (5 × 10^6^) were inoculated into the rear flanks of female nude athymic (NU/J) mice (Jackson Labs, Bar Harbor, ME, USA). Tumors were allowed to grow freely until they reached a size ≥ 0.75 cm. At this point, mice (four or more) were anesthetized with continuous flow (2.5%) isoflurane and placed inside lead coffins with only the tumor-bearing flank exposed within the radiation field. Mice were then irradiated with 2 Gy daily over 5 days for a total dose of 10 Gy (i.e., 5 fractions of 2 Gy) using a Small Animal Radiation Research Platform (SARRP) (Xstrahl, Inc., Suwanee, GA, USA) housed in the Radiation and Free Radical Research Core facility.

### 4.7. MRI Analysis

MRI analysis was conducted as previously described in [23,44]. Tumors were then imaged on a 7T GE MR901 small animal scanner, a part of the small animal imaging core. T2*-weighted images were collected using a gradient echo sequence (TR = 10 ms, TE = 2.2, 8.2, 14.2, and 20.2 ms, matrix = 256 × 256, FOV = 25 × 20 mm, 2 signal averages). A B0 shimming routine was performed to limit the effect of macroscopic field inhomogeneities. T2* maps were generated using a combination of 4 echo times collected and fitting each voxel to a mono-exponential curve using in-house Python 3.7.0 code. Images were imported to 3D Slicer software (V5.0.3), where regions of interest (ROIs) were delineated as a 1 mm diameter cylinder in the center of the tube, and mean T2* values were calculated using the label statistics tool within 3D Slicer.

### 4.8. Labile Iron Analysis

Labile iron concentrations in tissue samples were determined using a ferrozine-based colorimetric assay. Tissue samples were homogenized in a 1X RIPA lysis buffer (Sigma-Aldrich; R0278). Cell debris was removed by centrifugation at maximum speed (14,000× *g*) for 10 min. A total of 150 µL of the supernatant was then diluted with 150 µL ferrozine buffer (5 mM ferrozine, 1.25 M ammonium acetate, 10 mM ascorbate) in a single well of a clear 96-well plate. Following dilution, the 96-well plate was evaluated for the formation of a Fe^2+^–ferrozine complex by monitoring the absorbance at 562 nm. For statistical analysis, the absorbance was normalized to the tissue dry weight and then assessed relative to control samples.

### 4.9. Statistical Analysis

All experiments were performed with at least 3 replicates, with α = 0.05 being used as a threshold to determine statistical significance. Analysis was performed using GraphPad Prism v10.4.2 software.

## 5. Conclusions

As interest in the pro-migratory effects of ionizing radiation on glioma grows, it has become important to understand the mechanism(s) underlying such an effect. In this study, we have identified iron as a contributing factor to the enhanced cell motility observed following exposure to ionizing radiation, as radiation-induced TfR expression was associated with decreased cell stiffness. Importantly, the overexpression of FtH was able to reverse the enhanced cell motility following radiation. These findings underscore the potential importance of iron in regulating tumor cell migration at the physical level and open a therapeutic vulnerability avenue to exploit and enhance the management of GBM patients.

## Data Availability

The data supporting this article will be made available upon reasonable request by the editor.
